# Microglial Activation Alters Chromatin Accessibility of AD‐Associated Transcription Factors

**DOI:** 10.1002/alz.085990

**Published:** 2025-01-03

**Authors:** Zena K Chatila, Jason Mares, Badri N. Vardarajan, Wassim Elyaman, Vilas Menon, Elizabeth M Bradshaw

**Affiliations:** ^1^ Columbia University, New York, NY USA; ^2^ The Institute for Genomic Medicine, Columbia University Medical Center, New York, NY USA; ^3^ Department of Neurology, Columbia University, New York, NY USA; ^4^ Center for Translational & Computational Neuroimmunology, Department of Neurology, Columbia University Irving Medical Center, New York, NY USA

## Abstract

**Background:**

While dysregulated local innate immunity and microglial dysfunction are thought to play a pathogenic role in Alzheimer’s disease (AD), the underlying mechanisms remain unclear. Importantly, activation of immune and metabolic pathways in myeloid cells can lead to a functional reprogramming process, termed **innate immune memory (IIM)**, in which the response to an initial stimulus shapes long‐lasting epigenetic modifications that alter the response to future inflammatory stimuli. This epigenetic imprinting process has been minimally studied in microglia. We posit that immune activation may result in epigenetic reprogramming which contributes to sustained microglial dysfunction as a pathogenic mechanism in AD.

**Method:**

We treated an *in vitro* primary monocyte‐derived microglial model with a range of activators of specific innate immune receptors, disease‐associated stimuli, and pathogens. We investigated whether any of these stimuli induce epigenetic remodeling using single nucleus RNA‐and ATAC‐sequencing after a washout and seven‐day rest period, and probed for alterations in AD‐associated molecular pathways. We compared the rewired epigenetic signatures we observed to ATAC‐sequencing data from human microglia from post‐mortem AD brain tissue.

**Result:**

We found that three stimuli in particular resulted in unique remodeled chromatin signatures: *E. coli* LPS (TLR4 activator), CpG ODNs (TLR9 activator), and HSV‐1 infection. Motif enrichment identified binding sites of AD‐associated transcription factors within the differential chromatin peaks remodeled by these stimuli. For example, TLR9 activation with CpG ODNs altered chromatin accessibility of *MEF2A* and *MEF2C* binding sites, transcription factors that have been genetically associated with AD. We identified that features of the unique chromatin signatures associated with these stimuli overlap with post‐mortem microglial chromatin configuration from human AD brain tissues. Lastly, we identified the functional consequences of microglial IIM induced by these three primary stimuli upon delayed secondary stimulation, including altered cytokine response.

**Conclusion:**

Microglial activation by specific inflammatory stimuli causes epigenetic rewiring, resulting in differential accessibility of AD‐associated transcription factors and altered response to future stimuli. In this way, microglial activation and IIM may later contribute to microglial dysfunction in AD pathogenesis.

